# Evolved Resistance to Placental Invasion Secondarily Confers Increased Survival in Melanoma Patients

**DOI:** 10.3390/jcm10040595

**Published:** 2021-02-05

**Authors:** Yasir Suhail, Junaid Afzal, Kshitiz

**Affiliations:** 1 Department of Biomedical Engineering, University of Connecticut, Storrs, CT 06269, USA; yasir.suhail@uconn.edu; 2 Cancer Systems Biology (CaSB@Yale), Yale West Campus, West Haven, CT 06477, USA; 3 Center for Cell Analysis and Modeling, University of Connecticut Health, Farmington, CT 06032, USA; 4 Department of Cardiology, University of California, San Francisco, CA 94143, USA; Junaid.Afzal@ucsf.edu

**Keywords:** melanoma metastasis, stromal invasion, cancer dissemination, cancer associated fibroblasts, invasibility, placentation, cancer microenvironment

## Abstract

Mammals exhibit large differences in rates of cancer malignancy, even though the tumor formation rates may be similar. In placental mammals, rates of malignancy correlate with the extent of placental invasion. Our Evolved Levels of Invasibility (ELI) framework links these two phenomena identifying genes that potentially confer resistance in stromal fibroblasts to limit invasion, from trophoblasts in the endometrium, and from disseminating melanoma in the skin. Herein, using patient data from The Cancer Genome Atlas (TCGA), we report that these anti-invasive genes may be crucial in melanoma progression in human patients, and that their loss is correlated with increased cancer spread and lowered survival. Our results suggest that, surprisingly, these anti-invasive genes, which have lower expression in humans compared to species with non-invasive placentation, may potentially prevent stromal invasion, while a further reduction in their levels increases the malignancy and lethality of melanoma. Our work links evolution, comparative biology, and cancer progression across tissues, indicating new avenues for using evolutionary medicine to prognosticate and treat human cancers.

## 1. Introduction

Although humans and many other mammals are very vulnerable to melanoma metastasis, melanoma exhibits limited malignancy in many other mammals (e.g., cows and horses) [1]. Curiously, cancer malignancy rates among mammals are correlated with the extent of placental invasion into the maternal endometrium during pregnancy [2,3,4,5]. We recently explained this correlation, positing that endometrial fibroblasts in epitheliochorial species (with limited placental invasion) have evolved to acquire resistance to trophoblast invasion [6]. This acquired resistance is secondarily manifested in other stromal tissues, thereby limiting cancer dissemination in epitheliochorial species. This evolutionary framework, termed Evolved Levels of Invasibility (ELI) [6], can therefore guide the elucidation of mechanisms, which render stromal fibroblasts to resist dissemination of trophoblasts, or tumor cells—the first step in the metastatic cascade.

Stromal contribution to cancer dissemination, as well as to the progression of metastasis, is now well recognized [7,8,9]. Invasion into the stroma is the first step within the metastatic cascade, and for melanoma, stromal permeability of a lesion is strongly linked to malignancy [10,11,12]. In many cancer types, the stromal transcriptomic state has been linked to cancer dissemination and its progression to a metastatic disease [13,14,15,16].

Based on transcriptomic analysis of fibroblasts from multiple mammalian stromal tissue, we identified genes that change correlatively with the phenotype of increased (or decreased) placental invasion, purportedly also correlated with cancer malignancy [17]. Herein, we report a surprising finding, that the ELI-predicted gene expression trajectory derived from inter-species stromal differences is significantly correlated with malignancy outcome in human melanoma. Using patient-wide data from The Cancer Genome Atlas (TCGA) [18] and normal tissue transcriptomics from the Genotype Tissue Expression (GTEx) database [19], we found that the evolved determinants of stromal resistance showed a strong signal in human skin cancers, with the loss of expression in predicted anti-invasive genes linked to higher melanoma malignancy and low survival. We also identified key ELI-predicted anti-invasive gene signatures correlated with survival rates among human patients. Beyond providing further support for the ELI framework, this report underlines the relevance of evolution-inspired paradigms to further our understanding of the complex genetic trends in human pathology.

## 2. Results

### 2.1. Endometrial Genes Correlated with Placental Invasion Among Mammals

**Figure 1:**
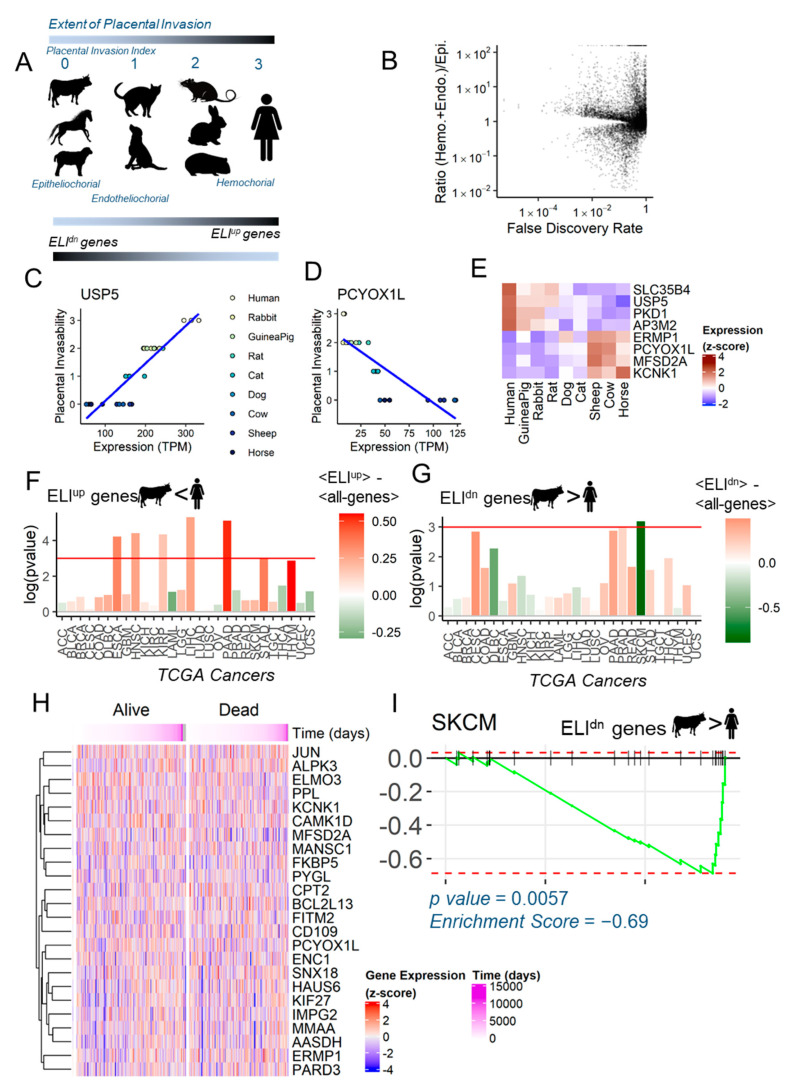
Evolved Levels of Invasability (ELI) in mammals and its signature in human cancer. (**A**) The extent of physiological placental invasion differ among depicted mammals, rated manually as shown between 0 and 3. Using a phylogenetic linear regression model, genes significantly correlated with increased invasion were termed ELI^up^, while those anti-correlated with invasion were termed ELI^dn^. (**B**) Global view of the genes and their statistical test for correlation to placental invasion (*x*-axis), and the ratio of expression in hemochorial and endotheliochorial species vs. in epitheliochorial species. (**C**,**D**) Examples of individual genes USP5 and PCYOX1L, whose expression across species (*x*-axis) is correlated (positively and negatively, respectively) with the extent of placental invasion (*y*-axis). (**E**) Heat map showing the relative expression (z-scores) of four ELI^up^ and four ELI^dn^ genes across species. (**F**,**G**) Overall differential expression of ELI^up^ and ELI^dn^ gene data derived from Gene Expression Profiling Interactive Analysis (GEPIA), respectively, in different cancers represented in The Cancer Genome Atlas (TCGA). T-tests were conducted for the difference between ELI^up^ or ELI^dn^ and all other genes in mean log fold change for cancer/normal. Multiple cancers show significant ELI^up^ overexpression, and melanoma (SKCM) shows ELI^dn^ under-expression. The height of the bars correspond to the statistical significance of the difference in fold changes of the ELI genes vs. the rest, and the color corresponds to this difference in means (red for increase in expression of ELI genes in cancer vs. normal, and green for decrease in expression). (**H**) Gene expression across patients of the top 25 ELI^dn^ genes. Patients were stratified based on their mortality until the last follow-up, arranged by time until death or last follow-up (if alive). (**I**) Gene Set Enrichment Analysis (GSEA) of the top 25 ELI^dn^ genes. All genes were ranked in order of their fold change in melanoma (SKCM) vs. normal, left to right. The 25 ELI^dn^ genes are shown as vertical tags on the top line. The green line is the running score that decreases for each gene not in the set, and increases for genes among the 25 ELI^dn^ genes. The 25 ELI^dn^ genes found to be enriched for under-expression in melanoma. Analysis and visualization is as described by Subramaniam et al. [21].

The evolutionary framework Evolved Levels of Invasibility (ELI) that we advanced links the large differences in placentation to malignancy rates among eutherian (placental) mammals [6]. For melanoma and other skin cancers, for which wider zoological data are available, epitheliochorial species with non-invasive placentation (e.g., cows and horses) show limited malignancy [1]. In contrast, hemochorial species (e.g., humans and rodents) exhibit highly aggressive placental invasion into the endometrium, and high rates of melanoma malignancy. ELI posits that the evolution of acquired maternal resistance to placental invasion in epitheliochorial species (e.g., cows and horses) is also manifested secondarily in other stromal tissues, leading to lower rates of malignancy [6]. To identify the genes that correlate with this phenotype, the Wagner Lab previously collected endometrial fibroblasts from multiple mammalian species [20], and together, we ranked them according to the invasiveness of their placentation (epitheliochorial species: cows and horses ranked 0; endotheliochorial species: cats and dogs ranked 1; hemochorial species: rats, rabbits, and guinea pigs ranked 2; particularly invasive primates: humans ranked 3) [17]. Using a linear phylogenetic model, we identified the stromal genes that had changed correlatively to the placental invasion phenotype. Genes that increased in expression in stromal fibroblasts of more invasive placentation (e.g., humans) were termed ELI^up^, while those that correlatively increased in non-invasive placentation (e.g., bovines) were termed ELI^dn^. Since ELI^up^ genes are up-regulated in more invasive species, these are purportedly pro-invasive, while ELI^dn^ genes being up-regulated in less invasive species are purportedly anti-invasive, according to the ELI framework (Figure 1A) [17]. The overarching hypothesis of this study was that ELI genes also show similar pro- or anti-cancer properties in melanoma patient samples. We found that among the 8000+ orthologs common to all species analyzed, more genes were up-regulated in hemochorial species vs. those in epitheliochorial species (Figure 1B). Figure 1C (*p*-value = $1.33\;\times 10^{-9}$, Pearson correlation = 0.90) and 1D (*p*-value = $5.98\times 10^{-7}$, Pearson correlation = −0.83) show an example of an ELI^up^ and ELI^dn^ gene each, correlatively changing in expression along with the placental invasion phenotype. A correlative change in expression among these species with different levels of stromal resistance (Figure 1E) can provide us with a guide to identify mechanisms driving stromal resistance to cancer dissemination.

### 2.2. Anti-Invasive ELI^dn^ Genes Are Negatively Enriched in Human Skin Cancers

We set out to test whether ELI genes were enriched in human cancers, and could therefore play a plausible role in the progression of metastasis. Because ELI is a stromal fibroblast centric framework, one ideally needs stroma specific transcriptomic data of human cancers to test the enrichment of ELI-predicted genes in cancers. Unfortunately, single-cell sequencing has only now picked up pace, and large databases of hundreds of human cancers at single-cell resolution may yet take time to be created. With this caveat, we asked if the current human cancer transcriptomic databases, e.g., The Cancer Genome Atlas (TCGA) [18], could be utilized to test the enrichment of ELI-correlated gene sets in either the cancer, or the normal tissue. We chose the top 25 ELI^up^ (purportedly pro-invasive) genes and the top 25 ELI^dn^ (purportedly anti-invasive) genes, and tested their relative differential expression in tumor vs. normal tissues for all of the represented cancers in the TCGA database. All raw gene expression numbers were normalized to z-scores using the mean of the normal tissue derived from the GTex database [19], as well as with the averaged fold changes for all genes in each cancer type. We found that ELI^up^ genes were indeed enriched in many cancer types, including esophageal, head and neck, kidney, pancreatic, and thymus and stomach cancers (Figure 1F). Interestingly, we found that in cancers of skin origin (SKCM), there was a significant reduction in the expression of ELI^dn^ genes (Figure 1G). More data exist about the correlation of placental invasion to melanoma, and our own experimental data showed that bovine skin fibroblasts resist melanoma invasion much more strongly than do human fibroblasts [6]. There was a substantial heterogeneity in gene expression for the 25 ELI^dn^ genes analyzed in the 468 human skin cancer samples in TCGA (Figure 1H). We therefore performed a gene set enrichment analysis (GSEA) to calculate if the purportedly non-invasive genes are differently enriched in skin cancer vs. normal samples (Figure 1I). GSEA analysis [21] showed that ELI^dn^ genes were likelier to be reduced in the expression in SKCM samples, with the leading genes containing PPL, a desmosomal protein called perplakin, KCNK1, a potassium ion channel component, CAMK1D, a key serine threonine kinase in the Ca^2+^/calmodulin signaling, JUN, a well-known proto-oncogene, and PARD3, a cell polarity regulator and an inhibitor of glioma invasion [22]. Notably, even though ELI^dn^ genes are expected to be expressed less in human stromal fibroblasts vs. bovine (albeit, endometrial fibroblasts), they may confer physiological resistance to invasion, and down-regulation of these genes may lead to lowered resistance to cancer dissemination. Since the comparison to normal tissue samples is based on normal skin only, while the SKCM dataset also includes melanoma-origin metastatic tissues from lymph nodes in addition to primary melanoma tissues, the cancer vs. normal expression differences may be somewhat mixed with inter-tissue differences.

### 2.3. Anti-Invasive ELI^dn^ Genes Are Associated with Increased Survival in Human Melanoma Patients

**Figure 2:**
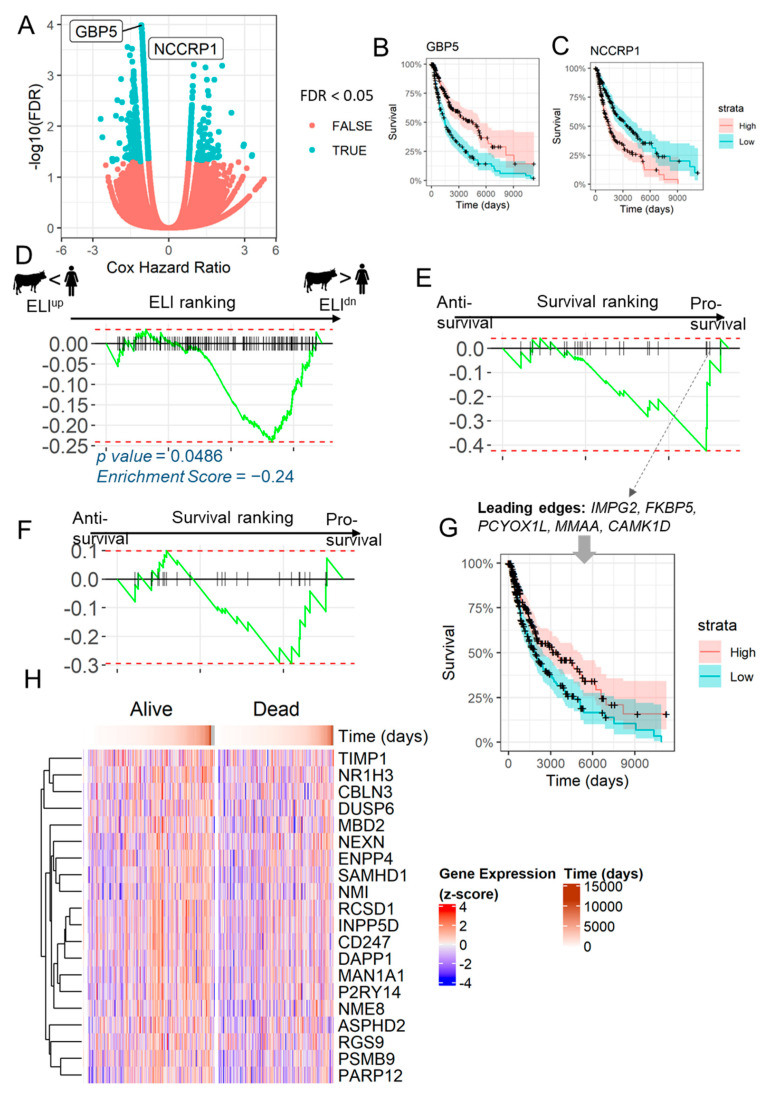
Gene-wise survival analysis reveals positive prognosis for ELI^dn^ genes in melanoma. (**A**) All genes were tested for their effect on survival using the Cox proportional hazard test, using patient data from TCGA’s SKCM project. Shown are the statistical significance (*y*-axis) and the hazard ratio (*x*-axis) for individual genes. Genes are colored by their false discovery rate (FDR). (**B**,**C**) Effect on survivability for the most significant pro-survival (GBP5) and anti-survival (NCCRP1) genes for their expression above and below the median expression in melanoma cases. (**D**) GSEA plot of the ranking 500 most pro-survival genes in the order of the calculated ELI correlation for all genes from inter-mammalian comparisons. Only genes with an ELI score (known unique orthologs across all species) were included. Pro-survival genes were significantly enriched for ELI^dn^ genes. (**E**) GSEA plot of the ranking of the 25 most ELI^dn^ in the order of their effect of survival prognosis. ELI^dn^ genes were significantly enriched for pro-survival genes. (**F**) GSEA plot of the enrichment of same 25 most ELI^dn^ genes, but in the order of the survival ranking using only patients with samples taken from skin, cutaneous, or subcutaneous tissues. (**G**) The top five genes selected from the leading edge of the GSEA plot in (**E**), and the effect of their average expression (median-normalized z-score) on survivability. (**H**) Heat map showing the expression (z-score) of 20 ELI^dn^, pro-survival genes across patients, with patient samples arranged in the order of their time to death or live follow-up.

As the stromal genes correlated with increased resistance to invasion were found to be lost in melanoma specimens, we asked whether the loss of expression of these gene is linked to patient outcome. We therefore mapped each gene expression to the survival data available for 471 SKCM cancer samples, thereby calculating the hazard ratio for each gene; from a total of 54,439 genes, 292 genes were found to be significant (*p* < 0.05) for anti-survival, and 1926 for pro-survival (Figure 2A). A positive hazard ratio suggests that the gene is pro-survival, for example, GBP5 (Figure 2B), while a negative hazard ratio indicates that the gene is deleterious and its increased expression reduces remaining life years, as shown for NCCRP1 (Figure 2C). We ranked genes according to their correlation with placental invasion, and tested the enrichment of pro-survival genes in melanoma (Figure 2D). GSEA analysis confirmed that survival is enriched in genes with a negative ELI ranking, the anti-invasive ELI^dn^ genes (Figure 2D). Then, we ranked all genes according to their calculated hazard ratio, and tested the enrichment of top 25 ELI^dn^ genes. GSEA analysis clearly showed that ELI^dn^ genes were significantly likelier to be pro-survival than anti-survival (Figure 2E). Although TCGA’s SKCM dataset only includes cancers of primary melanoma origin, a significant portion of the samples were resected from metastatic sites in lymph nodes (212 out 470). To verify if the effect of ELI genes is detected specifically in skin samples, we also ranked the genes by the survival signal (hazard ratio) using only the data from patients with tumors resected from skin and subcutaneous tissues in Figure 2F. The top 25 ELI^dn^ genes were also enriched for the pro-survival trait (as illustrated by the enrichment score of −0.3) in skin samples in Figure 2F, but with a slightly lower signal than in the analysis of all samples (with an enrichment score of −0.4 in Figure 2E). Although there was considerable patient-level heterogeneity for the leading edge genes from the GSEA analysis, when patients were ranked according to their survival beyond initial diagnoses, we found that the leading ELI^dn^ genes that were enriched in the pro-survival gene set were likely to be higher in longer-living patients (Figure 2G). The leading edge genes did confer increased survival among melanoma patients (Figure 2H).

### 2.4. Loss of Anti-Invasive ELI^dn^ Genes is Associated with Decreased Survival in Melanoma Patients

**Figure 3:**
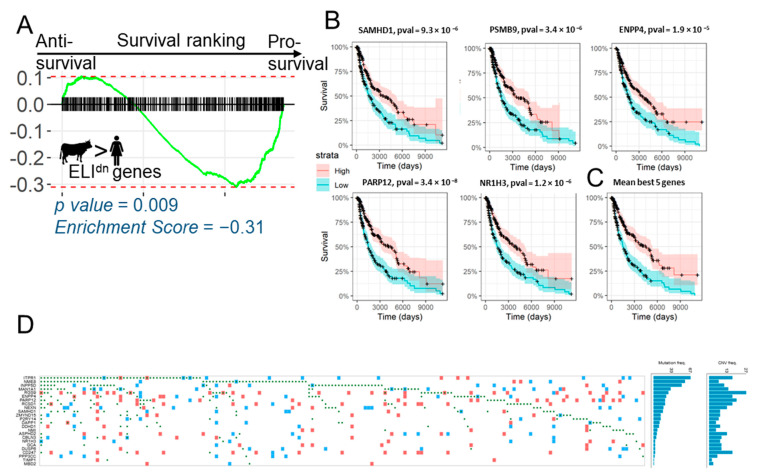
Loss of expression of ELI^dn^ genes reduces melanoma patient survival. (**A**) GSEA plot of the ranking of the top 500 ELI^dn^ genes in the order of their effect of survival prognosis. ELI^dn^ genes are significantly enriched for pro-survival genes. (**B**) Five of the most pro-survival genes from the leading edge of (**A**) are shown, with their effect on survivability, with the averaged effect (median-normalized z-score of their expression) shown in (**C**,**D**) Mutation and copy number variations in the ELI^dn^, pro-survival leading edge genes from Figure 3A. Figure generated from TCGA Oncogrid. The green dots correspond to single nucleotide polymorphisms, and red and blue squares correspond to the gain and loss in copy number variation, respectively.

Our analysis showed that, remarkably, gene changes correlated with evolution of epitheliochorial placentation, and which ELI framework posits to be likely anti-invasive, are lost in human melanoma patients, and also their loss is correlated with reduced survival rates. To further confirm that loss of ELI^dn^ genes in melanoma reduces survivability, we ranked genes according to the calculated hazard ratio, and tested for the enrichment of all epitheliochorial ELI^dn^ genes (Figure 3A). GSEA analysis showed that ELI^dn^ genes were indeed enriched toward the pro-survival axis (Figure 3A), as they were for a smaller gene set (Figure 2E). We identified the top five leading edge genes, which were most pro-survival in human melanoma patients, and were also anti-correlated with placental invasion. All top five genes, with the possible exception of ENPP4 with no previous report on its role on metastasis, have been reported to be anti-metastatic in different cancer types. NR1H3 is a nuclear receptor component and an anti-invasive gene for bladder cancer [23]; PARP12 has been reported to suppress hepatocellular carcinoma metastasis [24]; SAMHD1 is yet another suppressor of epithelial transformation [25]; PSMB9 is a key metastasis suppressor for breast cancer [26]. We tested these leading edge genes for their effect on the survival of human melanoma patients. We found that, individually and together, these leading edge gene signatures indicated a significantly increased survival of skin cutaneous melanoma patients (Figure 3B,C). These data, particularly in the light of no signal from ELI^up^ genes in SKCM enrichment, suggest that, surprisingly, it is the loss of anti-resistive signals that leads to both melanoma progression and low survival in patients. Finally, we explored how the reduction of expression in these genes may occur from existing genomic information in TCGA, showing the occurrence of single nucleotide polymorphisms and copy number variations in the pro-survival genes in 186 mutated cases in Figure 3D. We found that for the top 25 leading edge pro-survival genes enriched in the ELI^dn^ gene set, a few coding sequences had mutations or copy number reductions. Genes where mutations were present in a fraction of the population were ITPR1, a gene encoding an intracellular receptor for the lipid signaling molecule, IP3, releasing Ca^2+^ from the endomplasmic reticulum upon ligand binding; NME8 encoding a thioredoxin domain-containing gene; as well as another gene in the lipid signaling family, INPP5D, encoding the inositol polyphosphate−5-phosphatase. Therefore, it appears that non-genomic regulation explains most of the expression differences of these genes in cancer vs. normal tissue, which could either be due to secondary regulation, or because these genes may be stromal in origin, and therefore less plausibly subjected to genomic changes.

## 3. Methods

### 3.1. Data Sources

Gene expression profiles of endometrial stromal fibroblasts across species, and unique ortholog mappings from 8639 genes were obtained from the Wagner Lab [6] (NCBI Bioproject PRJNA564062). Gene expression fold changes between cancer and normal tissue for different cancer datasets were obtained from the Gene Expression Profiling Interactive Analysis (GEPIA) [27] tool. Patient-wise tumor gene expression and clinical data were extracted from TCGA’s SKCM project (https://portal.gdc.cancer.gov/projects/TCGA-SKCM). The mutation and copy number variation were obtained from TCGA’s Oncogrid.

### 3.2. Statistical Analysis

Statistical analysis was conducted using custom scripts on the R platform [28]. Gene scores for the Evolved Levels of Invasibility (ELI scores) were calculated using the Pearson correlation test. *p*-values were adjusted for multiple testing correction using the false discovery rate method. Top genes for ELI^dn^ and ELI^up^ were selected by ranking the most negative and positive correlation coefficients.

Student’s t-tests were conducted for differences in the mean fold changes of ELI genes vs. all other genes. Statistical significance (*p*-values) and effect sizes (differences in means) are reported in the figures.

Cox proportional hazard model tests [29] were conducted using the survival package [30,31]. For each gene, the gene expression was converted to a binary variable signifying expression greater or lower than the median across all melanoma samples. To show the combined effect of multiple genes (Figure 2G and Figure 3C), firstly, robust z-scores for the genes were computed by subtracting the median and dividing by the median absolute deviance. Then, the mean z-score was separated into the high (z > 0) and low (z < 0) strata.

All Gene Set Enrichment Analysis (GSEA) followed the standard Kolmogorov–Smirnov tests, and were conducted using the fgsea package [32]. For ranking by ELI scores, the correlation coefficient was used as the order statistic. For ranking by the survival signal, we converted the *p*-value of the test into a z-score, with the sign taken from the sign of the hazard coefficient. Thus, pro- and anti-survival genes were ranked in the order of statistical significance. Visualization of GSEA followed the method of Subramaniam et al. [21]. We used the term leading edge to denote the subset of genes most extremely ranked up to the point where the running score reaches the maximal absolute value. For gene sets with overall enrichment of high ranked genes, this corresponds to the most highly ranked subset whose members were all higher ranked than expected by chance.

## 4. Discussion

Stromal contribution to progression of malignancy is now well recognized, although a systematic understanding of the genetic and non-genetic bases for stroma-induced malignancy is still lacking [7,9]. It is well recognized that fibroblasts are altered in a tumor microenvironment, which could render part of their population pro-inflammatory and part pro-invasive [7,33]. Although single-cell sequencing data are now becoming available for many cancer types, it is still too scant to ascribe gene-to-phenotype relationships for fibroblasts [34,35]. Furthermore, there are large transcriptomic heterogeneities in the stromal state of patients, making it difficult to obtain gene expression signatures that inform the pro- or anti- behavior of cancer-associated fibroblasts. Our evolutionary framework, ELI, attempts to provide a partial answer to the mechanisms of stromal resistance, or the lack thereof, leading to the onset of malignancy.

The recent evolution of epitheliochorial placentation with its characteristic non-invasive placentation, according to ELI, is partly due to an evolved stromal resistance to trophoblast invasion [6]. This resistance is secondarily manifested in other tissues, resulting in lowered malignancy rates, particularly for melanoma, for which wider zoological data are available. We also experimentally demonstrated that bovine skin fibroblasts reduce stromal invasion of a variety of human melanoma cell lines. In this study, we attempted to find translational evidence of these evolved genes as anti-metastatic genes in human melanoma patients.

Our analyses indicate that it is the loss of ELI^dn^ genes in melanoma correlating with the evolution of non-invasive epitheliochorial placentation that confers decreased survival in patients. Because these genes are expressed higher in epitheliochorial species vs. humans [17], it is interesting that these genes continue to confer increased protection against melanoma progression and patient survival. An important caveat is that a stromal-specific signal for each gene cannot be delineated from bulk RNA sequencing data in TCGA. Therefore, it is plausible that these genes are either stroma expressed, or even if expressed in cancer cells are anti-metastatic in general. This heterogeneity of cell types in bulk tissues is likely to be different in different sites of cancer. We posit that the leading edge genes identified in this study could be effective therapeutic targets to enhance stromal resistance to melanoma dissemination, potentially limiting cancer malignancy. Because, the stromal cellular state differs among patients due to heredity, age [36], senescence [37,38], chronic inflammation [39,40], obesity [41,42], etc., an understanding of the stromal contribution to the progression of melanoma could be instructive in prognosticating disease spread, and in identifying therapeutic targets to limit melanoma malignancy. Finally, we note in this study gene set of predictions obtained through comparative biology across multiple mammalian species could be informative in understanding certain aspects of human disease in a tissue different from where the primary data were derived (skin and endometrium), squaring the circle between evolution, development, and human disease manifestation, and highlighting the connections in biology across different scales.
